# Case studies about smart contracts in healthcare

**DOI:** 10.1177/20552076231203571

**Published:** 2023-10-08

**Authors:** Cristina Vargas, Miguel Mira da Silva

**Affiliations:** Instituto Superior Técnico, 72971 University of Lisbon, Lisboa, Portugal

**Keywords:** IoT, IoMT, blockchain, smart contracts, ethereum, security, healthcare, and access control

## Abstract

The Internet of Things (IoT) such as devices and sensors are a fast growth reality which our bureaucratical and archaic institutional system is not yet ready to embrace its functionalities. In the health system, many developments are made, and smart devices are the key to preventing, studying, investigating, and solving a lot of diseases and improving our health system. But along with this, innovation is necessary for the hospitals, for example, to have a proper system that provides storage of health data information and respects the General Data Protection Regulation (GDPR) with the use of smart contracts that secure the integrity and disclosure of the patient's data, since the majority of hospitals still use paper, physical records to store data. In this study, we will briefly analyse and explain three different suggested methods to deal with the challenges that Internet of Medical Things (IoMT) encounters. We will not choose which one is the best because of the different features and the countries they are proposed but will emphasize the benefits and challenges which one has.

## Introduction

In the fast world of technologies, Internet of Medical Things (IoMT) is providing smart tools to prevent and monitor patients’ conditions, use medical devices that will allow doctors to have access to sensitive medical data, and update and transmit the data. COVID has increased even more the importance of digital health technologies.^1–3^

But all that must be executed securely and flawlessly to avoid vulnerabilities in smart equipment and protect privacy data from attacks (smart hospitals may encounter malware, ransomware, and tampering with medical devices) that can be ‘just’ stealing important data or even worse cause a patient's death!

To provide high security to users, it has been accepted and it's mentioned in several studies that the blockchain technology working along with smart contracts^4,5^ in its many variants is the most effective source for IoMT data security. Blockchain's decentralized^6–11^ platform is tamperproof due to its cryptographic technology used to authenticate users in the network and a decentralized^12–14^ storage system can also provide low-cost off-chain storage to store supply chain transaction data.

Another health area where smart contracts and decentralized off-chain storage show efficiency and results is in pharmaceutical supply chains, to avoid counterfeit and ensure product safety. The use of smart contracts on supply chains can guarantee data provenance, makes the use of intermediaries unnecessary, and provides a secure, immutable history of transactions to all stakeholders.

It is very important to provide an efficient framework to establish security for IoT devices because weak security can facilitate access to patient health records. The electronic health record (EHR) is the most important and sensitive category of data because it contains very intimate, private, and classified information related to the patients and their diagnoses.^15^

In this study, we will analyse three systems that work with blockchain, block structures based on Ethereum,^16–18^ and smart contracts where it can be used a fog-cloud or machine learning for example.

The discussion isn’t around what is the best one or the most effective for a health system/institution but to start realizing the different options and purposes that those systems can provide. Monitoring diseases, real-time updates of the patient status, possible communication^19^ between applications, medical devices or machines, and institutions, to provide clear information, help to prevent future diseases, decrease the health system costs, and secure storage^20^ and transactions.^21^ Of course, the more complex and effective health technology is, the more expensive it can become.

However, adapting the needs to the specific system may save millions for the health system. IoMT brings important discussions around patients’ personal and sensitive data, which each country must be prepared to adapt to its legal system.

Smart contracts play an important role in the consent, storage, and legality of data processing. The national legal system must be able to follow the technological evolution, accept it, and adapt to it by creating new legislation and/or updating the legislation already implemented.

We will explain what is blockchain and smart contracts, by initially saying that the sequence of chains on a blockchain, in addition to the information in each block, will have an identifier, called a hash, and another hash associated with the immediately preceding block in the chain (except the first block obviously). The hash will be a fingerprint that identifies your block and its order in the chain of blocks; in this sense, block 2 will have the hash that allows identifying block 1, used in blockchain technology right from the start because it makes it more secure.

**Figure 1.**
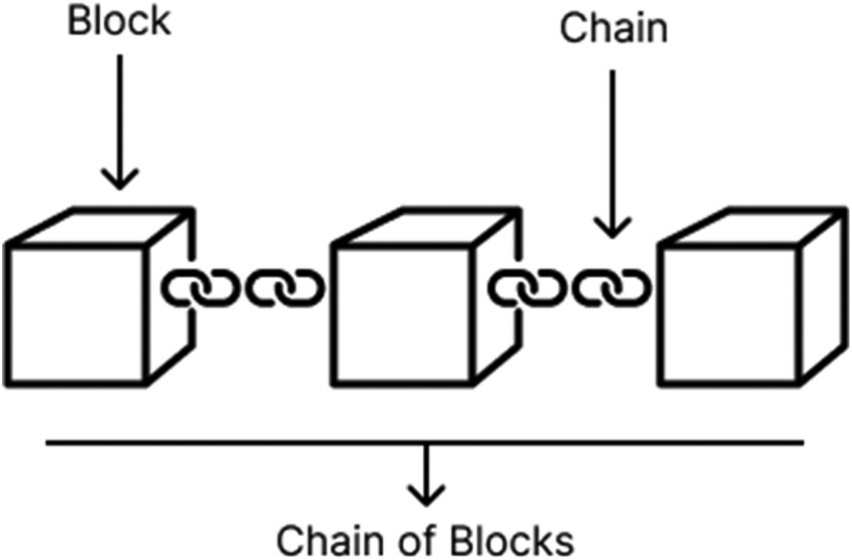
Blockchain.

The blockchain works as a set of rules defined in a programming language called Protocol, which is created by the platform creator (Figure 1).

Blockchain technology has evolved since Bitcoin^22–24^ and is now considered a new form of distributed ledger as all data can be stored in transaction metadata. Due to this possibility, many rely on the blockchain health-related applications that are displayed quickly. Example applications include immutable patient data, improved healthcare data sharing^5^/analysis without loss of control, and improved reliability of counterfeit drug^25^ detection/prevention systems in pharmaceutical supply chains.^26^

Other applications using blockchain technology, contract management systems, and digital content distribution systems have been developed. Several blockchains have been created, such as purchase and sell agreement.^27^

Smart contracts are automated agreements of will, in a codified way so that they are easily fulfilled and the execution of the contract is controlled.

**Figure 2.**
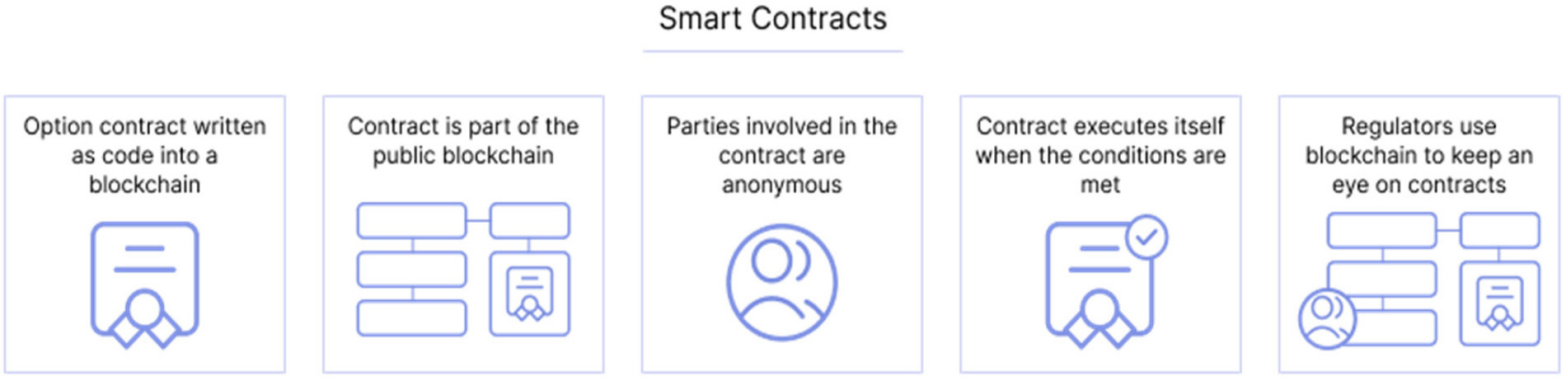
Structure of smart contracts.

There are applications that run on a custom blockchain, similar to Bitcoin.^28^ Ethereum's ability to develop smart contracts makes it possible to implement complex applications such as financial exchanges and insurance contracts on a distributed platform^27^ (Figure 2).

Smart contracts are created and exist within the Blockchain in the following way:

1. The terms of the contract are defined between the parties in the form of a code;
2. The contract is executed when the instruction stipulated by the parties is implemented;
3. There is no need for intermediation;
4. The contract is sent to the Blockchain that controls the execution of the contract, the code (the so-called smart contract code), and the Blockchain users;
5. The contract is registered on the Blockchain.

This makes it possible to eliminate the ambiguity of contracts in general, increase efficiency in their execution, and substantially reduce the risk of default between the parties. It also increases the transparency, integrity, and immutability of contracts.

As for the notion of smart contracts, it differs. If it is a broad notion, it refers to the smart contract code (code used in the Blockchain that executes certain instructions and allows computers to perceive them) or the more restricted notion that is the smart legal contracts (agreement of wills between the parties aimed at the automated production of certain legal effects, such as the common contract).

And that is why smart legal contracts (which use smart contract code) are a more restricted subtype of the smart contract code concept, as it can be used for several purposes, one of which is the creation of contracts that can be self-executed on the Blockchain.

It eliminates the need for human confirmation of precursors and outcomes, instead relying on machines that automatically follow programme-defined logic to accept or reject the request.^26^

The definition created by Nick Szabo is hybrid as it includes both notions explained, first referring to the smart contract code when referring to the protocol (a computerized protocol) and then referring to the smart legal contracts (which execute the terms of a contract).

There are situations in which, to be able to execute the smart legal contract, it is necessary to attend to events external to the Blockchain, and to that extent, oracles are used (oracles are independent entities that send information from the outside world, information that is essential for the Blockchain: they can be hardware oracles, software oracles, inbound oracles, or consensus-based oracles for example).

The Internet of Things (IoT) refers to the rapidly growing network of connected objects that are capable of collecting and exchanging data in real time using embedded sensors, e.g. thermostats, cars, lights, and refrigerators, connecting to a person's heart rate, patient by the pacemaker, smart homes, vehicles, and other devices that can be connected to the IoT.

Legally, we have the issue of data immutability on the Blockchain, which apparently can conflict with the right to erase data and the right to be forgotten, provided for in terms of the European General Data Protection Regulation (Regulation (EU) 2016/679 of the European Parliament and of the Council of 27 April 2016.

And still without resolution is the decentralization of this technology, or being given the capacity and legal personality to the Blockchain.

In the domain of liability of programmers (who may or may not be users), users, and limitation of liability of legal persons that operate in it, there is still a long legislative path to go which will be interesting to develop in the next paper (legal application and civil liability of smart contracts).

Blockchain technology and smart contracts are excellent tools for automating and enhancing the management and storage of health data. The use cases for these technologies are changing along with them. One significant way that smart contracts can be utilized to enhance people’s lives is through healthcare applications.

Up to 10% of deaths in the USA may be caused by medical errors, according to *Johns Hopkins Medicine*. Thousands of lives could be saved annually if smart contracts could be utilized to improve patient medical record data, drug supply chains,^29^ and medical collaboration.

**Figure 3.**
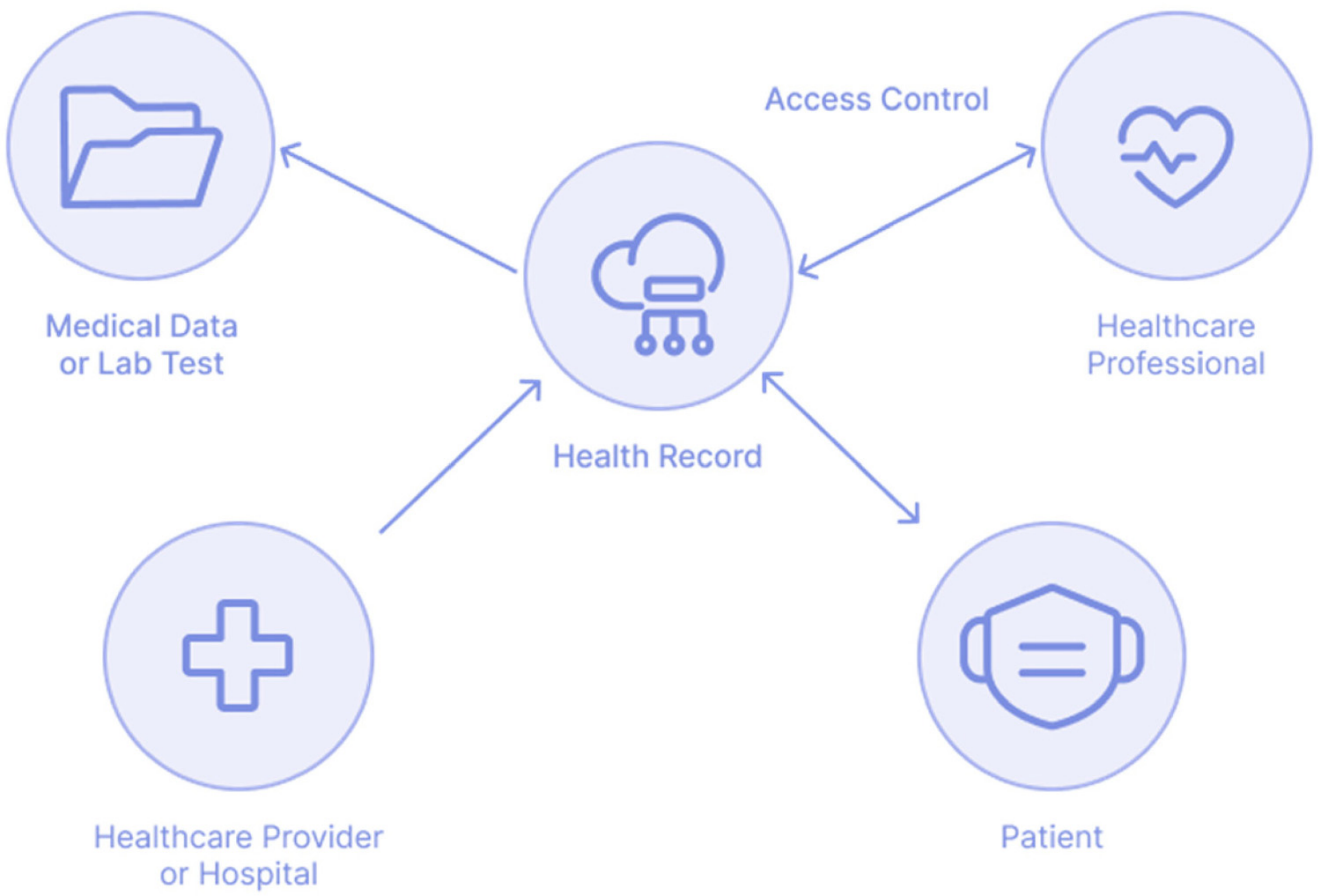
Based on ‘overview of the current system’.^32^

Distributed ledgers and public blockchain are innovative new technologies that enable access to and enhancement of medical research data and patient records^30,31^ for healthcare practitioners. The immutable, encrypted nature of smart contracts can improve patient privacy while enabling service providers to follow rules (Figure 3).

## Research methodology

In this paper, the authors searched all available articles that had any relation with the research we were focused on: valid* or legal* or regul* and ‘smart contract*’ and blockchain* and health. We obtained 136 articles total that had any content on their abstracts regarding those themes.

We exported them to https://www.rayyan.ai/ where we had automatic and more efficient access to the abstracts. After that, we eliminated all articles that were duplicates and were left with 102 articles. Of those 102 articles, we read all the abstracts to see which ones had the content we were looking for. We chose 53 articles that had interesting abstracts for our search. So we read the articles to find which ones were more interesting for us. That could answer our questions. So, we found 40 articles. Of those 40 articles, we made a more profound reading and chose 3 blockchain systems that were created for health system data conservation.

Unfortunately, we did not find articles regarding the legal issues that each may be confronted with, so that would be an interesting subject to exclusively develop in the next article. The questions we were looking at were the following:

- Can we create a blockchain system using smart contracts for health systems?
- Is it blockchain system secure?
- Does it provide access and control to the patient?
- Can the patient's information safely circulate from hospitals and institutions?
- Is the information storage regularly updated?
- Are these systems expensive?

On each of the three analysed systems, we can have our questions answered and see that some of them are concerned with data storage, others with data circulation between users, and others concerned with the security of that data storage. And we will begin to explain each system.

## IoMT with Ethereum using Blockchain in fog-cloud

Blockchain with IoMT using flog-cloud is where is proposed a smart contract with function-based cost-efficient task scheduling (FTS-CSON) algorithm in a blockchain framework. The main goals are to reduce the costs of the transactions, during data offloading and scheduling applications, and make them more secure.

A stronger basis for coordinating remote resources and performing tasks is provided by the cloud with a fog architecture (FA) built on blockchain technology. IoMT powered by fog computing is currently a prominent topic.^33^

**Figure 4.**
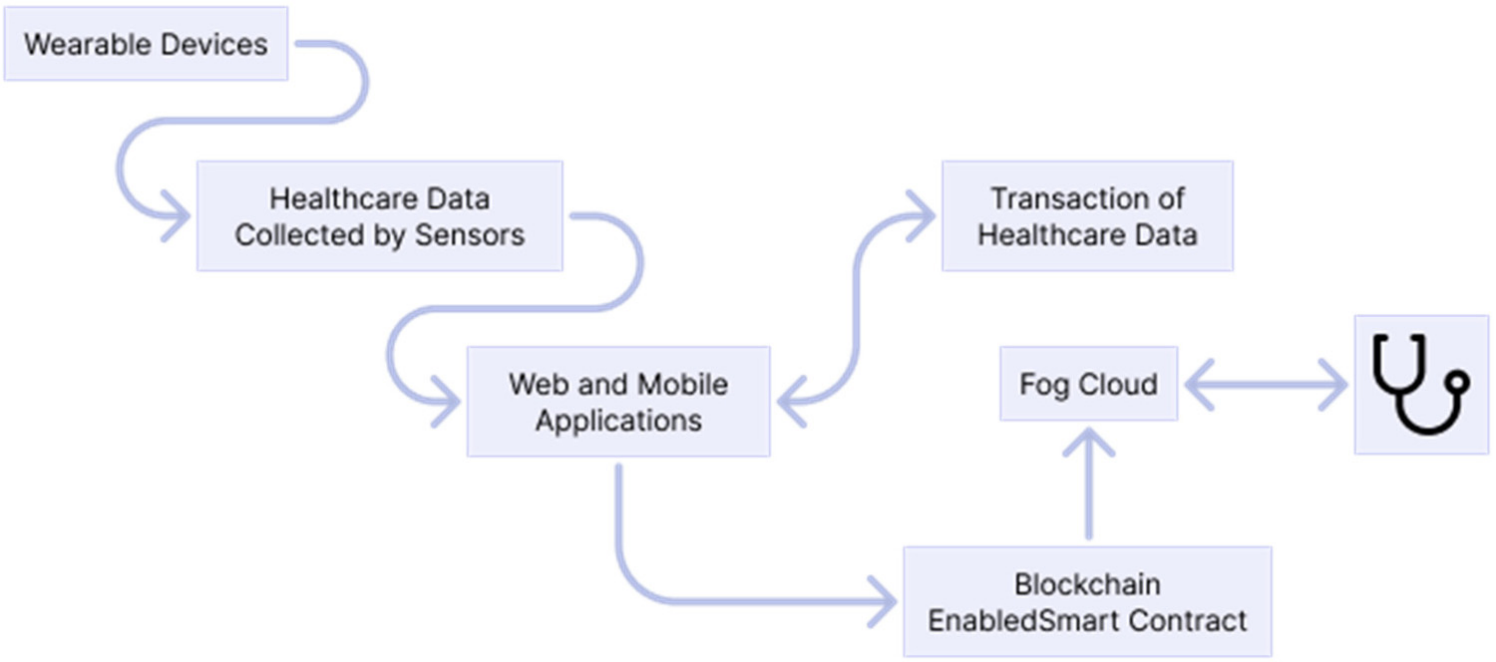
Based on ‘overall architecture of FTS-SCON’.^38^

Each task has a deadline to be completed with the threshold in the workflow. The cost-efficient is made using the IoMT application; the name given is blockchain-enable smart-contract cost-efficient scheduling algorithm framework (BECSAF) and will have the following steps:

1. The selection of each node connecting in the IoMT system is made by a smart contract rule, and each function has a different execution cost.
2. The security requirement of functions is identified by the function verification pool method before there is any addition to the system pool.
3. It generates a task and function sequencing.
4. It will generate a matching of resources (Figure 4).

The system will use smart electronic devices, such as smart belts, wearables, and medical/vital monitors, chest straps, among others, that will detect, analyse, and transmit the information needed through health application services.

The health sensors will have the function to store all the data on the blockchain-enabled smart contract and on the fog-cloud servers so they can perform the tasks. The fog-cloud server has to collect all the data and has fog-cloud nodes, which assist to process all data requests in the IoMT system, and at the end will transmit it to the physician.

In this architecture, smart contracts are created by Ethereum, because is an open-source and decentralized blockchain^2^ approach that allows user to create their own regulations and smart contracts. The group of data blocks on Ethereum are well structured, cannot be altered, and register all the transactions in the files.

This type of blockchain provides security^34–37^ for medical data, and the smart contracts are used for anyone or anything that wants access, performing a function of accessing control, that detects, tracks, and controls the data sharing.

**Figure 5.**
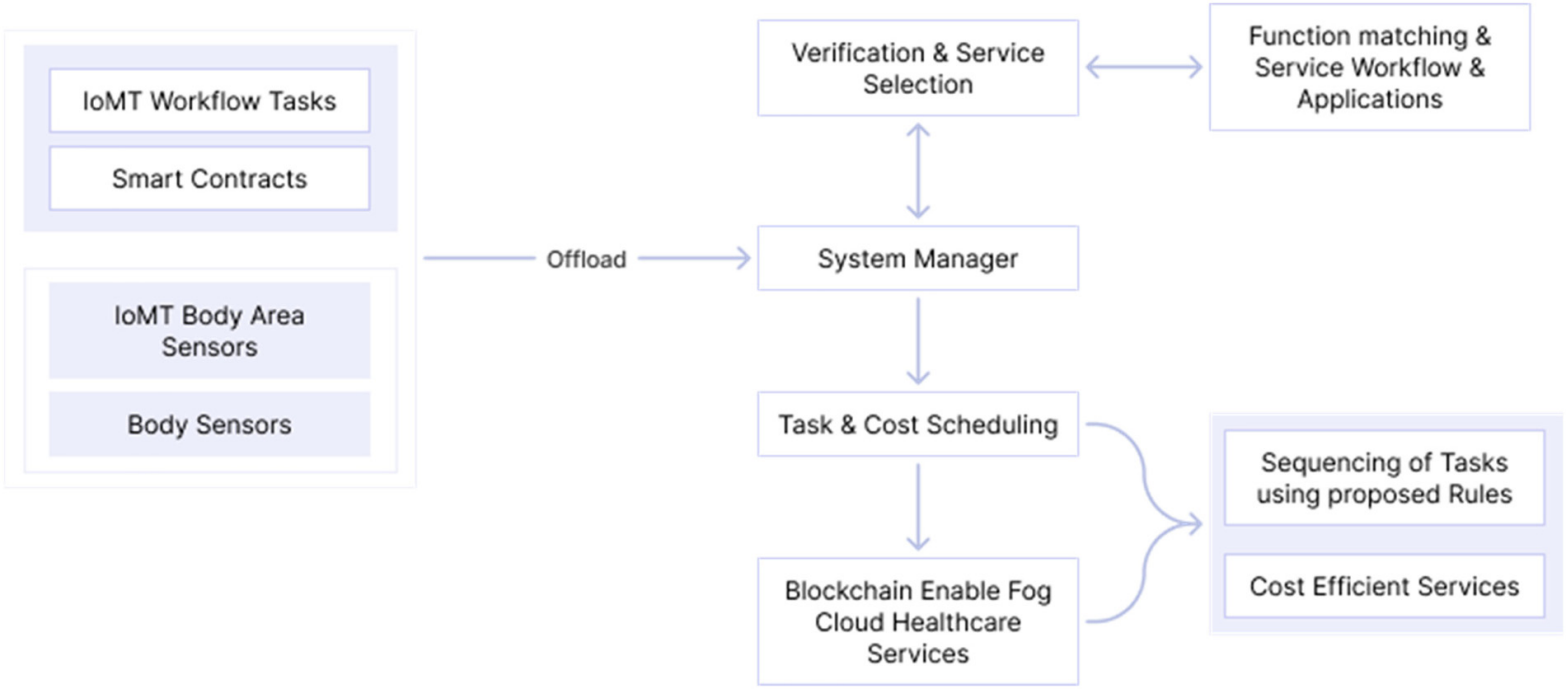
Base on ‘task and cost scheduling method and smart contract enabled blockchain network for IoMT’.^38^

In the following figure is the proposed method of FTS-SCON algorithm: workflows that connect several sensors and offload tasks to the blockchain (transaction is verified using blockchain technique) and enable fog-cloud healthcare servers for execution (Figure 5).

**Figure 6.**
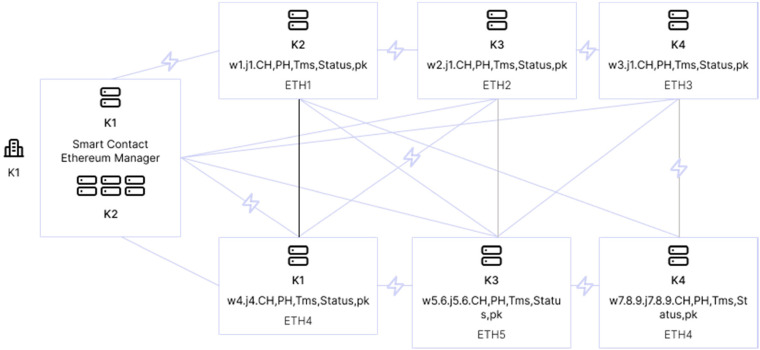
Base on ‘smart contract Ethereum mechanism in client flog cloud’.^38^

Figure 6 is explained how the transaction process executes all the tasks based on smart contracts, by a node. A public key is used for the data encryption, and the data-sharing wi and wz are validated by proof of work protocol.

In the research that used an Arduino heart rate monitor and a heartbeat sensor, the main conclusion was that the functions and their characteristics determine the cost of application execution. Nevertheless, the proposed system is proved by the authors to be less expensive in the health application system and gives more accuracy compared to the function based task scheduling blockchain-enabled framework (FTSB) and BECSAF systems and has more computation time. And not less importantly, the security risks are lower because the data is stored in the fog-cloud-enabled Ethereum.

## GuardHealth blockchain

The following study proposed a consortium blockchain-based smart healthcare system for health data privacy preservation, named GuardHealth.

The use of smart contracts that are arranged on Blockchain provides secure and efficient data storage and data sharing (basically consists of two smart contracts and a trust system to ensure, and provide security and privacy).^39^

The system separates raw data storage and index of data storage, and data are encrypted and stored in cloud service providers (CSPs). The user can allow requestors to access data and cancel permissions whenever necessary using proxy re-encryption. It also allows the user to make a profit through sharing data.

The system is considered trusty and improves the reliability of sharing data, and it also created a novel graph convolutional network (GCN) model to discriminate malicious nodes.

**Figure 7.**
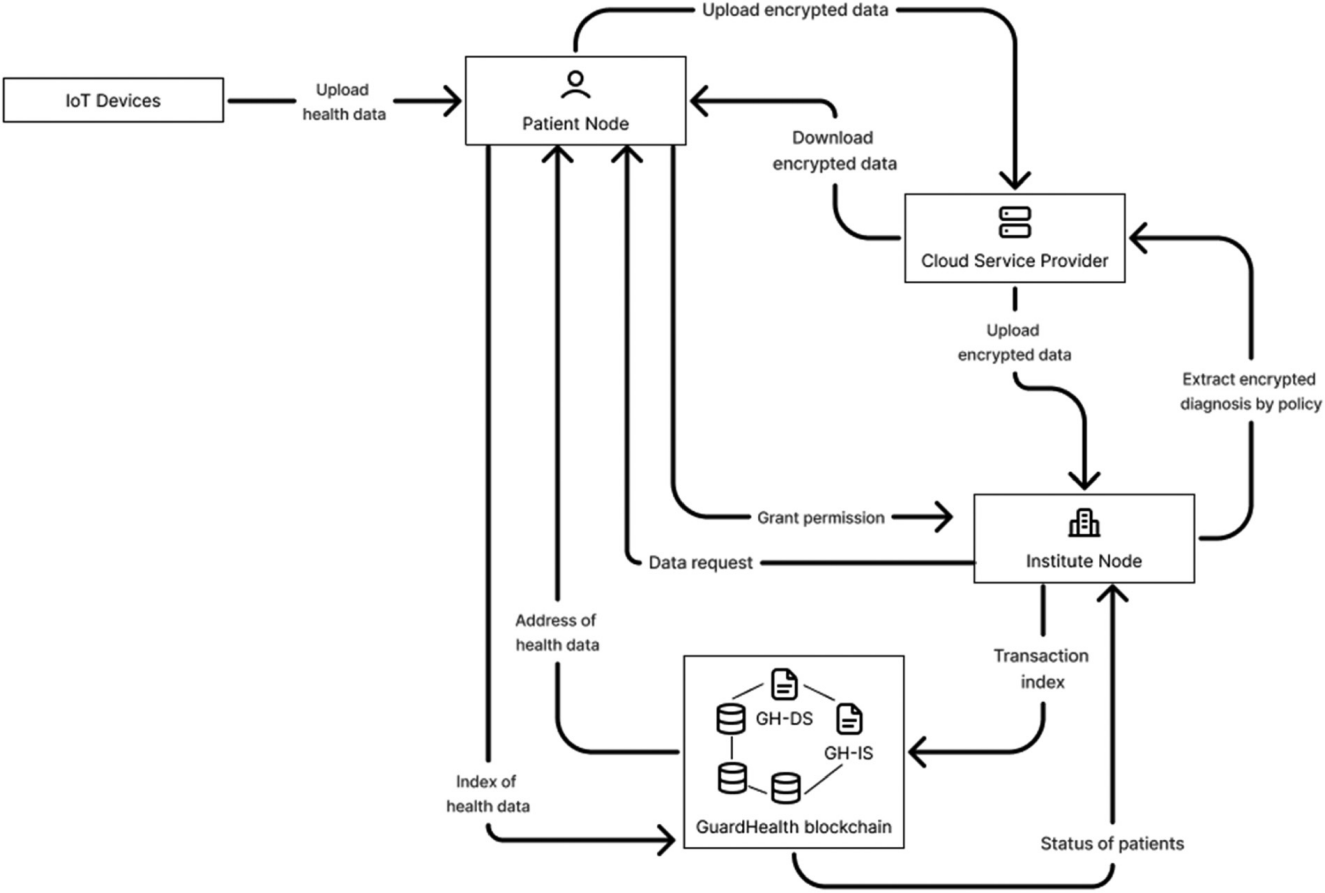
Base on ‘overview of the GuardHealth blockchain system using consortium blockchains’.^40^

The GuardHealth scheme has different characteristics which will be explained below (Figure 7):

**IoT devices** used to monitor different human health parameters (in wearable or implanted devices) collect the data and send the data which will be managed to patient nodes; these **patient nodes** store the data received from the IoT devices but also from hospitals or healthcare centres and do an important task which is to select the data and organize it by different security levels and can store a replica of their own encrypted data on other nodes (like CSPs) The patient nodes use symmetric encryption and encrypt the data which can only be decrypted by a specific patient.

**The CSP** will also store encrypted personal health data and data on GuardHealth chain (the CSP has a layer with the implementation of Kademlia with added persistence using LevelDB. The CSP is capable of carrying out several tasks such as diagnosis for uses using the health data from the sensor and institutes if they have the permission to do it.

The institute nodes consist of technology and pharma companies or hospitals, and they request the health data from patient nodes so they can make the analysis, but they are restricted by the rule of consortium, and only the authorized nodes can read or write health information updating the status of patient nodes.

Finally, the GuardHealth chain is also based on a consortium, so is a consortium chain that is used to update the status of user's health, and transaction data and access to it are only with permission. As it is on the blockchain system, GuardHealth chain is a series of blocks that contain the hash of a previous block and in this particular case contains transactions and the user's health status.

In the figure above, the user nodes upload the indexes of health data as the health status of the user on a chain with the regulation of GH-DS, a smart contract for data storage. The user's permission is required and determined so that the institute nodes can get access to the user's health data.

When it diagnosed the status of the user, it will be only updated after institutes update health data. On the other hand, if the institute nodes make a data request, it will activate the smart contract for data sharing, the GH-IS, and will establish an auction to pay for data from users and will generate transactions. The study recognizes the fragilities of CSP to attacks.

**Figure 8.**
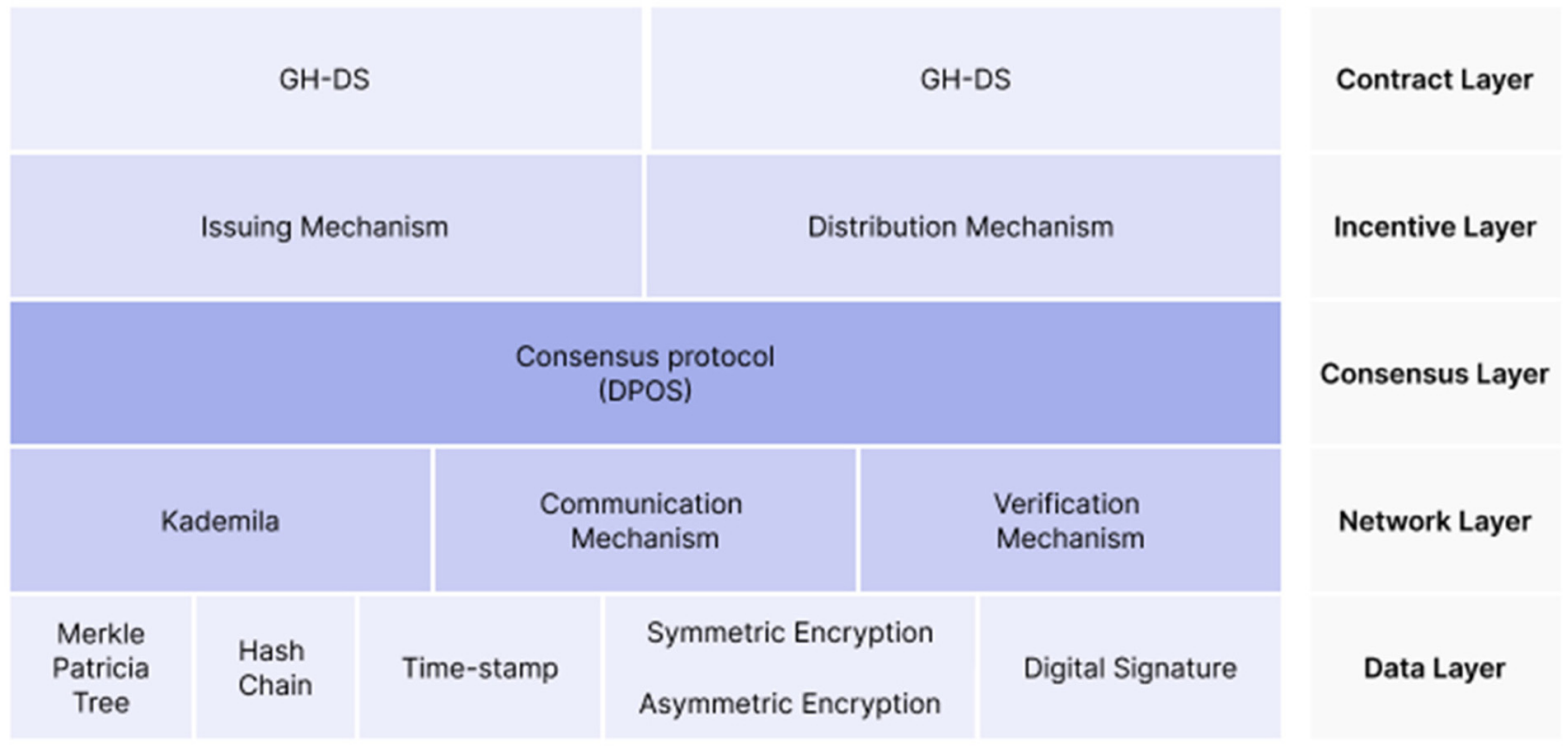
Based on ‘architecture of GuardHealth’.^40^

The architecture of GuardHealth provides a better and faster way of finding files using a distributed Hashtable in a distributed system when compared to an unstructured distributed system. The consensus layer makes a big difference to audit data before adding data to the chain. It inserted a delegated proof of stake (DPoS) to develop the consensus process, as you can see in Figure 8.

To make the distribution of data storage and data sharing among the parties and CSPs, a smart contract technology of two parts is used: smart contract GH-DS which defines the law of data storage (contains raw data and data Block) and GH-IS which defines the law of data sharing (transaction request, down payment, and data permission). These two smart contracts are autonomous, self-executed, and self-maintained among the users.

The system used is decentralized data that are encrypted and stored in CSPs or on their own devices, and the blockchain only contains the index encrypted data; the data are accessible only with the owner's permission.

Also in this project, the block structure is based on Ethereum. Not going on with technical details (data trust scheme used, detect malicious nodes by GCN, or Python simulation results), it is concluded that this system is more secure and efficient to preserve health data based on blockchain compared to the traditional schemes, although the malicious node detection model used was not very adequate.

## Machine learning

With the main focus on security data and cyber-attacks, this study develops a novel machine learning and blockchain technology-enabled framework capable of detecting cyber-attacks against health applications and at the same time allows international patient information and health data exchange.

This system presents a multi-layer healthcare-centric security information and event management (H-SIEM) framework with machine learning and big data analytics, reputation mechanisms, and visual-aided intrusion detection and prevention system (IDPS).

Permissioned blockchain technology is utilized to enable cross-border medical information sharing which allows several medical users access.

The solution found was security-enhancing and distributed access control (SEDAC), so is an improvement of the security of SIEMs with big data analytics, reputation algorithms, and visual-aided IDPS so it can detect any incidents that SIEM was not able to detect because SIEM systems have a limited capacity of dealing with network heterogeneity, and it does not have real-time prevention or detection techniques.

With SEDAC, all health devices are analysed so that anomalies can be traced. And blockchain technology in the form of smart contracts ensures privacy, security, and distribution of immutable information shared between hospitals in different areas and countries.

**Figure 9.**
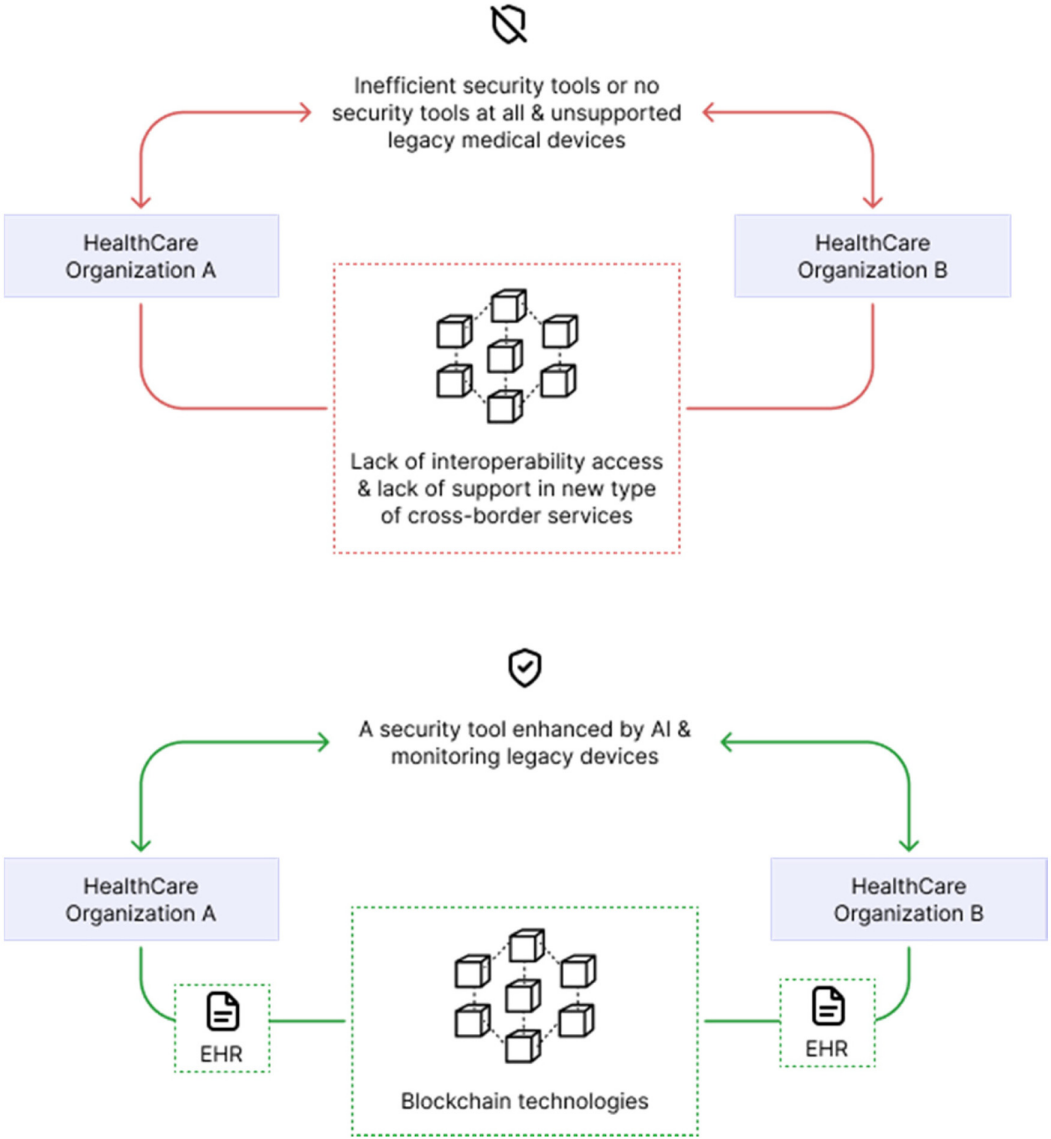
Base on ‘benefits of integrating SEDAC in healthcare’.^41^

The smart contracts provide the conditions so that this exchange of information can be done, and all transactions are registered on the distributed ledger. Again, the strong cryptographic nature of blockchain is important and necessary for SEDAC (Figure 9).

**Figure 10.**
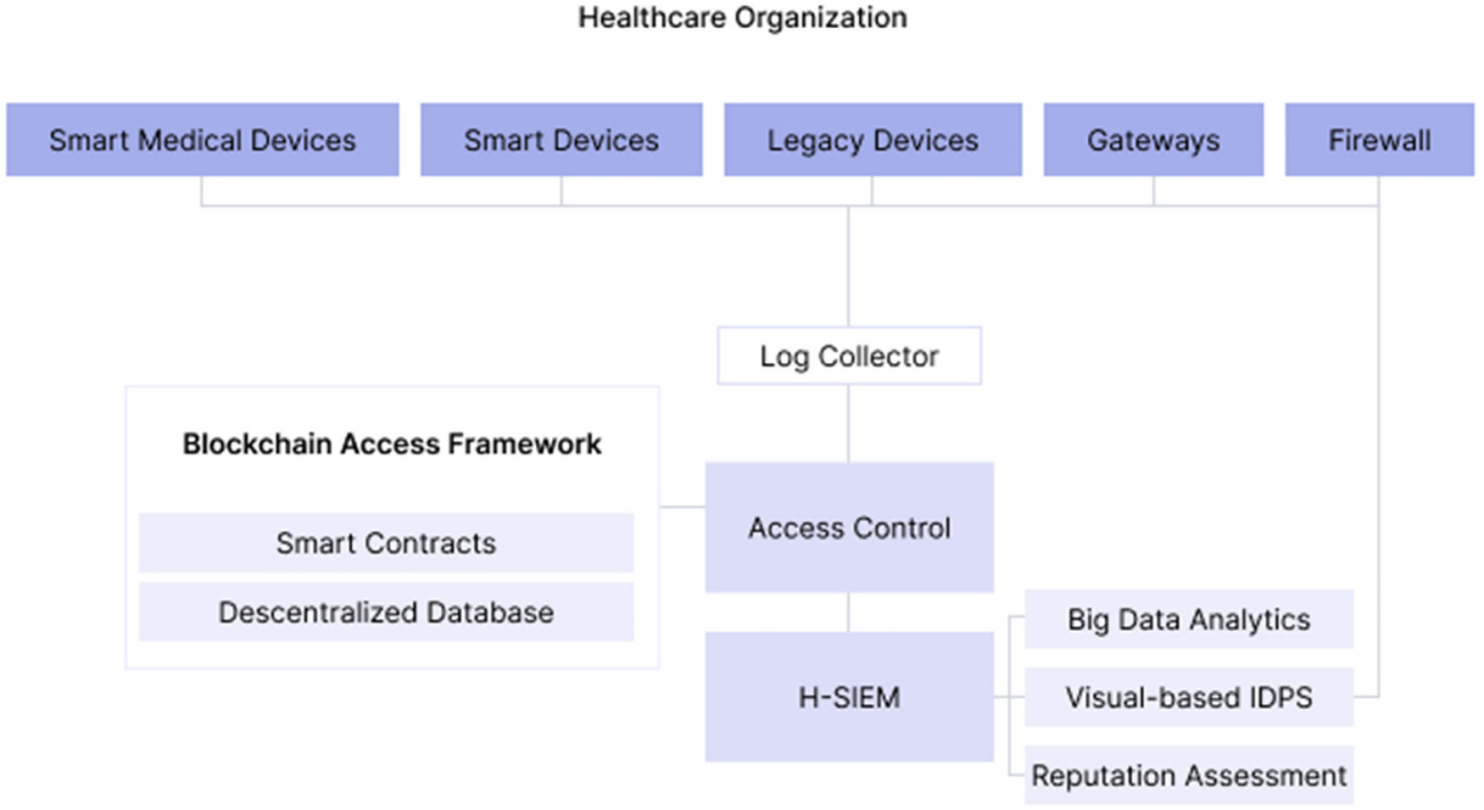
Base on ‘SEDAC framework architecture’.^41^

The security solution is H-SIEM which can monitor all system events (network traffic and system log information). Also, in this case, Ethereum smart contracts are used to access control, and it combined the role-based access control (RBAC) model with RBAC-SC to make user authentication and authorization which makes access to data possible until a certain level and will allow cross-border and cross-institution information to be exchanged and its architecture in the following way (Figure 10).

For SIEM to make informed decisions about possible threats, the adoption of a four-layer healthcare-centric SIEM framework named H-SIEM that prevents and detects threats, anomalies, and attacks against healthcare quickly is suggested.

So on the base layer is an open-source log collector, OSSIM SIEM (aggregates logs and network traces from everywhere, normalizes them, and produces alerts), and the second layer is big data analytics (that will apply machine learning to the collected data: logs, for example, so it can locate possible anomalies), the third layer is a reputation system (for the location of internal threats, using reputation algorithms), and the fourth layer is visual-based IDPS that combines the results of machine learning layer with reputation system layer and allows to detect any malicious acts.

**Figure 11.**
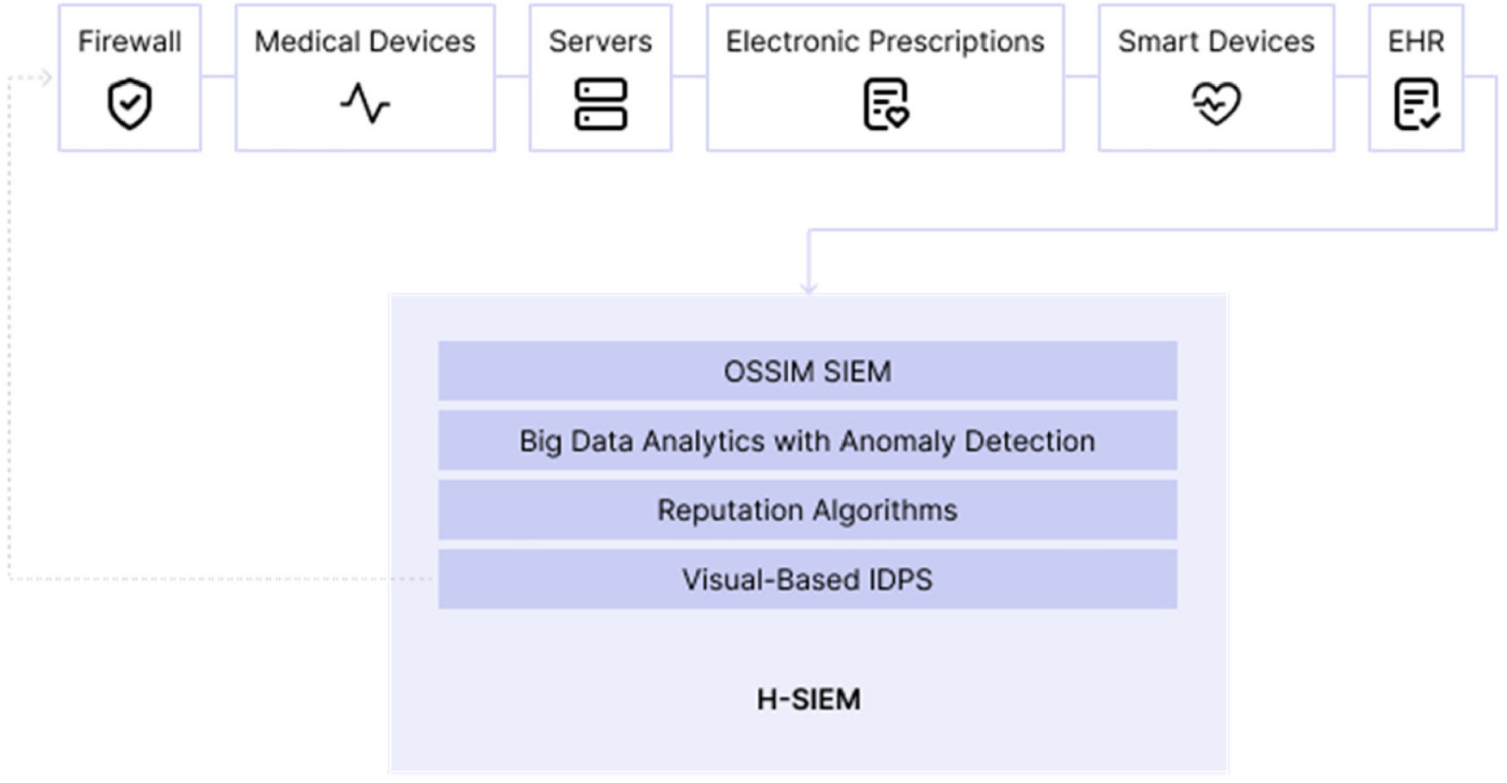
Base on ‘four-layer H-SIEM framework’.^41^

When threats are found, the IDPS will attempt to mitigate the attack by suggesting to the operator various countermeasures such as node isolation as we can see below (Figure 11).

Permissioned blockchain is again considered an important addition because patients should always authorize and have control of their health information and must be always according to the GDPR.^42^ Permissioned blockchain can enable access control through smart contracts, and only verified users can access the data all over the country, for example.

The study also criticizes the system normally used in hospitals; the RBAC is not efficient because of the constant evolution of the IoT devices^43^ and does not respect the GDPR requirements and finds this solution, H-SIEM, more viable and effective to secure the healthcare sector.

## Conclusion

The three systems presented and all the studies analysed for this paper have the same starting point: blockchain and smart contract and Ethereum platform. We believe Ethereum is currently the most suitable platform. The main reasons for choosing Ethereum were the ability to create a permissionless and a permissioned blockchain network, as well as the community development of the platform.^26^

Ethereum's ability to develop smart contracts^44,45^ allows you to run complex applications like financial exchanges and insurance contracts on a distributed platform.^27^

The two characteristics that nowadays are the most important are security of the medical and sensitive data and the reduction of costs. Any good system must provide these two features in a way that the users may make safe transactions (through the smart contracts implemented).

The IoMT will endure, and the more developed it is, the more attacks may occur. My big concern is the profit that data can generate and the illegalities that can be committed in favour of that profit, so if profit is inevitable, at least, has to have its legal boundaries. On the other hand, users have to understand how and where are the data being shared or stored and for how long, so the smart contracts implemented are crucial for the suitability of the transactions, and patients, for example, must always authorize the share of their data and control it.

A good security feature on the system and good legislation may prevent data violations. Important fact: European Commission just released on September 2022 a proposal for a regulation on a European health data space to improve individual's access and control to their electronic personal data and at the same time facilitate data reuse across the European Union.

The proposal creates rules, infrastructures, and mechanisms that allow primary and secondary use of electronic health data and introduces a mandatory self-certification scheme for EHR systems.

Although it is still in a very early stage, the EU has this concern and is beginning to create this legislation, and hopefully, all the EU countries involved will improve the health data space among main Europe and align with the GDPR legislation.
